# Assessing the effects of mining projects on child health in sub-Saharan Africa: a multi-country analysis

**DOI:** 10.1186/s12992-022-00797-6

**Published:** 2022-01-31

**Authors:** Hermínio Cossa, Dominik Dietler, Eusébio Macete, Khátia Munguambe, Mirko S. Winkler, Günther Fink

**Affiliations:** 1 Swiss Tropical and Public Health Institute, Kreuzstrasse 2, Allschwil, 4123 Switzerland; 2 University of Basel, P.O. Box, CH-4003, Basel, Switzerland; 3 Manhiça Health Research Centre, Maputo, 1929 Mozambique; 4 National Directorate of Public Health, Ministry of Health, Maputo, 264 Mozambique; 5 University Eduardo Mondlane, Faculty of Medicine, Maputo, 3453 Mozambique

**Keywords:** Child morbidity, Child mortality, Demographic and health survey, Diarrhoea, Mining, Nutrition, Sub-Saharan Africa

## Abstract

**Background:**

The African continent hosts many industrial mining projects, and many more are planned due to recent prospecting discoveries and increasing demand for various minerals to promote a low-carbon future. The extraction of natural resources in sub-Saharan Africa (SSA) represents an opportunity for economic development but also poses a threat to population health through rapid urbanisation and environmental degradation. Children could benefit from improved economic growth through various channels such as access to high-quality food, better sanitation, and clean water. However, mining can increase food insecurity and trigger local competition over safe drinking water. Child health can be threatened by exposure to mining-related air, noise, and water pollution. To assess the impact of mines on child health, we analyse socio-demographic, health, and mining data before and after several mining projects were commissioned in SSA.

**Results:**

Data of 90,951 children living around 81 mining sites in 23 countries in SSA were analysed for child mortality indicators, and 79,962 children from 59 mining areas in 18 SSA countries were analysed for diarrhoea, cough, and anthropometric indicators. No effects of the launch of new mining projects on overall under-five mortality were found (adjusted Odds Ratio (aOR): 0.88; 95% Confidence Interval (CI): 0.68–1.14). However, activation of mining projects reduced the mortality risk among neonates (0–30 days) by 45% (aOR: 0.55; 95% CI: 0.37–0.83) and risk for a child to develop diarrhoeal diseases by 32% (aOR: 0.68; 95% CI: 0,51–0.90). The timing analysis of observed changes showed that there is a significant decline in the risk for childhood diarrhoea (aOR: 0.69; 95% CI: 0.49–0.97), and the mean height-for-age z-scores by 28 percentage points, during the prospection and construction phase; i.e., within four years to the initiation of extraction activity. No effects were found for cough and weight-for-height.

**Conclusion:**

The results presented suggest that the impacts of mining on child health vary throughout the mine’s life cycle. Mining development likely contributes positively to the income and livelihoods of the impacted communities in the initial years of mining operations, particularly the prospection and construction phase; these potential benefits are likely to be at least partially offset by food insecurity and environmental pollution during early and later mining stages, respectively. Further research is warranted to better understand these health impacts and to identify policies that can help sustain the positive initial health impacts of mining projects in the long term.

**Supplementary Information:**

The online version contains supplementary material available at 10.1186/s12992-022-00797-6.

## Introduction

The African continent holds one-third of global natural resources [1, 2] and hosts more than 2000 industrial mining projects at different development stages [2, 3]. This number might further increase with the growing demand for various minerals to promote a low-carbon future [1]. While the extraction of natural resources represents an opportunity for countries rich in natural resources in sub-Saharan Africa (SSA), the impact of large-scale mining projects on the health of young children remains unclear.

On the one hand, mining projects can positively influence determinants of health and, thus, improve child health. For example, the development of mining projects has the potential to increase the share of workers with regular incomes – including women of reproductive age [4, 5] – and, thus, improves households’ capacity to buy healthier foods, access health care, and send the children to school [5–7]. Furthermore, mining projects can improve housing conditions, including proper sanitation and safe water [8, 9]. In turn, better housing, sanitation, and water conditions can reduce the incidence of environment-related diseases such as respiratory infections, diarrhoeal diseases, malaria, and undernutrition [10–12].

On the other hand, it has been reported that mining activities can have adverse effects on child health and development [13, 14]. For example, mining activities can negatively affect local and regional agricultural production through environmental degradation and changes in land use [15, 16]. Consequently, food insecurity can increase, which is of particular concern for young children and pregnant women [6, 17, 18]. Additionally, mining projects have high energy and water demand, potentially triggering local competition over existing resources, including access to safe drinking water [19–21]. In the contexts where natural resources are extracted, adverse environmental impacts such as air, noise, and water pollution are a significant concern for child health [20, 22, 23]. Studies found that exposure to environmentally poor conditions during the early stages of human life, including in-utero exposure, can result in long-term adverse effects on cognitive abilities, respiratory functions, and nutritional status [14, 17, 24].

Estimated impacts of mining projects on child health outcomes, such as diarrhoea, respiratory infections, and child mortality, have been highly heterogeneous to date [6, 25, 26]. One reason for the high heterogeneity seen in the empirical literature is the often differential focus on early (opening phase) vs. late (extraction phase) of mining [27, 28]. It also seems plausible that the heterogeneity of the currently available results is due to the narrow focus of current studies either on just one country or one mineral (such as gold) or both [6, 7, 14, 29].

This paper aims to understand the impacts of mining activities on child health using data from 81 mining projects launched across the sub-Saharan African region between 2002 and 2019. More specifically, we pursued the following research questions: (i) What is the effect of mine opening on child morbidity and mortality in sub-Saharan African countries? (ii) How many years before or after the launch of extractive activities can health impacts be detected?

## Methods

### Data sources and management

This study was conducted by combining two different georeferenced data sources, namely: (i) the socio-demographic and health data from Demographic and Health Survey (DHS) and (ii) mining data from the Standard & Poor’s Global Market Intelligence (S&P GMI) Mining Database [3]. Both data sets were restricted to SSA.

#### Socio-demographic and health data

**Fig. 1:**
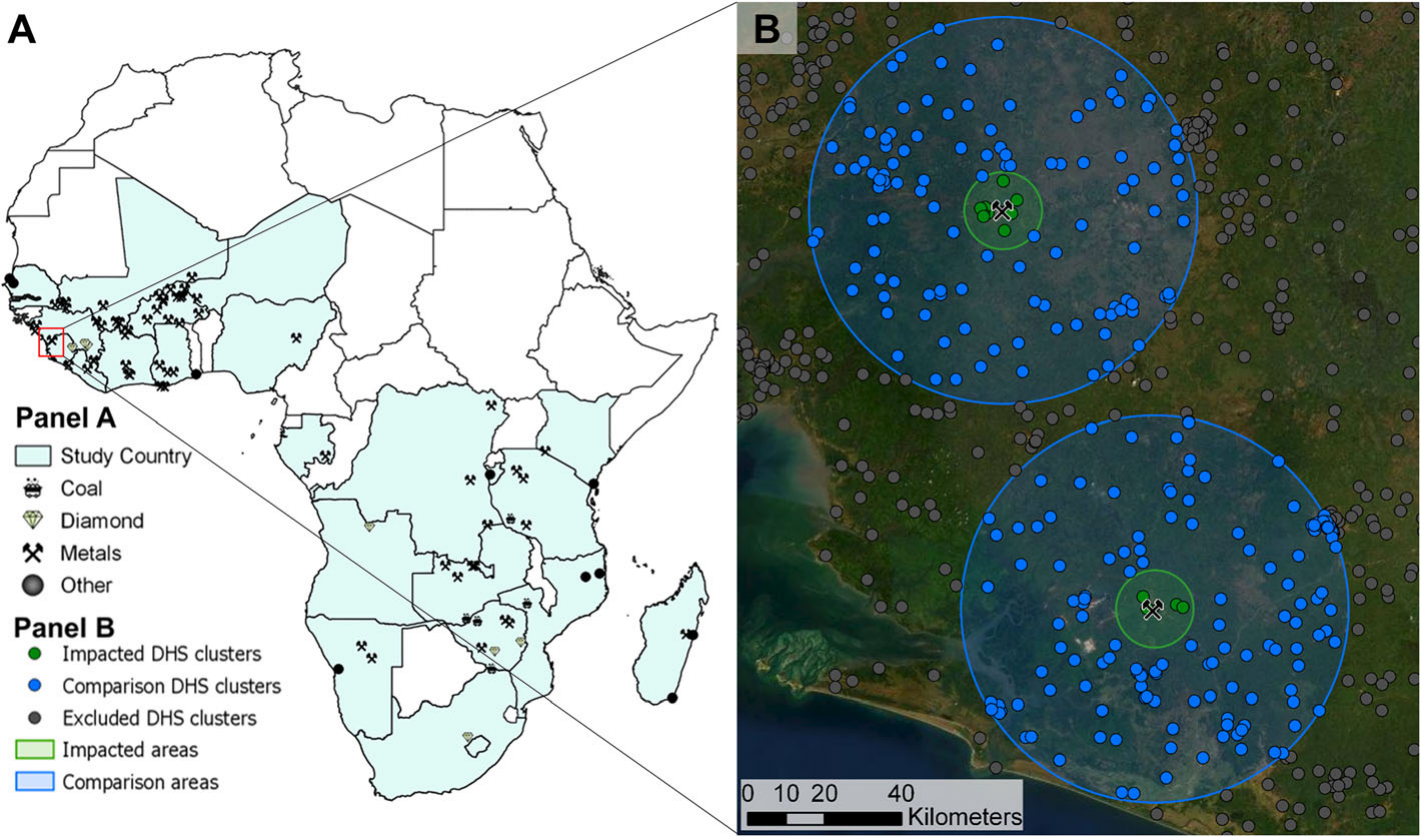
Spatial distribution of mines (panel A) and visualisation selected DHS clusters (panel B)

The DHS program conducts nationally and regionally representative household survey data in over 70 low- and middle-income countries. The DHS surveys are conducted following a two-stage cluster random sampling strategy, randomly selecting households within randomly selected enumeration areas. In most countries, DHS surveys are conducted every 4–6 years. The survey datasets are available on request on the website of the DHS program (www.dhsprogram.com). For this study, we use data from all DHS standard surveys from SSA for which geographic coordinates were available as of March 2020 (see Fig. 1, panel A). All household and child datasets were combined with the corresponding geographic data to merge with the mining data. Of note, the DHS program introduced random noise to the cluster coordinates to ensure the privacy of the respondents: in urban settings, clusters’ coordinates are shifted up to 2 km (km), and in rural areas, clusters are typically displaced by 5 km.

#### Mining data

The proprietary mining dataset was accessed through a subscription to the S&P Global Market Intelligence platform (www.spglobal.com) [3]. The mining data comprises four primary indicators: geographic point location (Global positioning system, GPS) coordinates, extracted commodities, and historic mining activities between 1980 and 2019 (e.g., mine opening and closure years). We set the year of mine activation (i.e., initiation of exploration and evaluation activities) at 10 years before the reported extraction onset, i.e., the earliest year of the operation phase with reported extraction or production. We did this, aiming to include the prospection and construction phase of the project. We created a sub-sample of mines that opened within the period during which DHS data were available (i.e. 1986–2019). Finally, mines located closer than 20 km from another mine were excluded to avoid overlapping impact areas (see Fig. 1, panel B). Panel A of Fig. 1 shows the 81 mines analysed by primary commodity extracted (coal (*N* = 5), diamonds (*N* = 7), metals (*N* = 59), and other mines (*N* = 10)).

#### Merging of datasets by spatial analysis strategy

The GPS coordinates for each DHS survey cluster and the mine point locations were used to match all surveyed households and children to one or several mines. DHS clusters within 50 km of the distance of each mine were selected. Based on previous studies showing that impacts are centralised within 10 km from a mining, project we set the treatment group within this distance range [4, 6–8, 14, 17, 30, 31]. Hence, clusters within 10 km from the mine were classified as “impacted clusters” (or treated), while clusters at 10–50 km distance were classified as “comparison clusters” (or controls). To assess the impact of mine opening events on child health outcomes, we restricted our analysis to mines with DHS records before and after the mine opening year. Figure 1 exemplifies the selection of data around mining projects in Sierra Leone. Figure 2 summarises the overall data set construction process. Data merging was done using ArcGIS Pro (Version 2.2.4, Environmental Systems Research Institute, Redlands, CA, USA).

**﻿Fig. 2:**
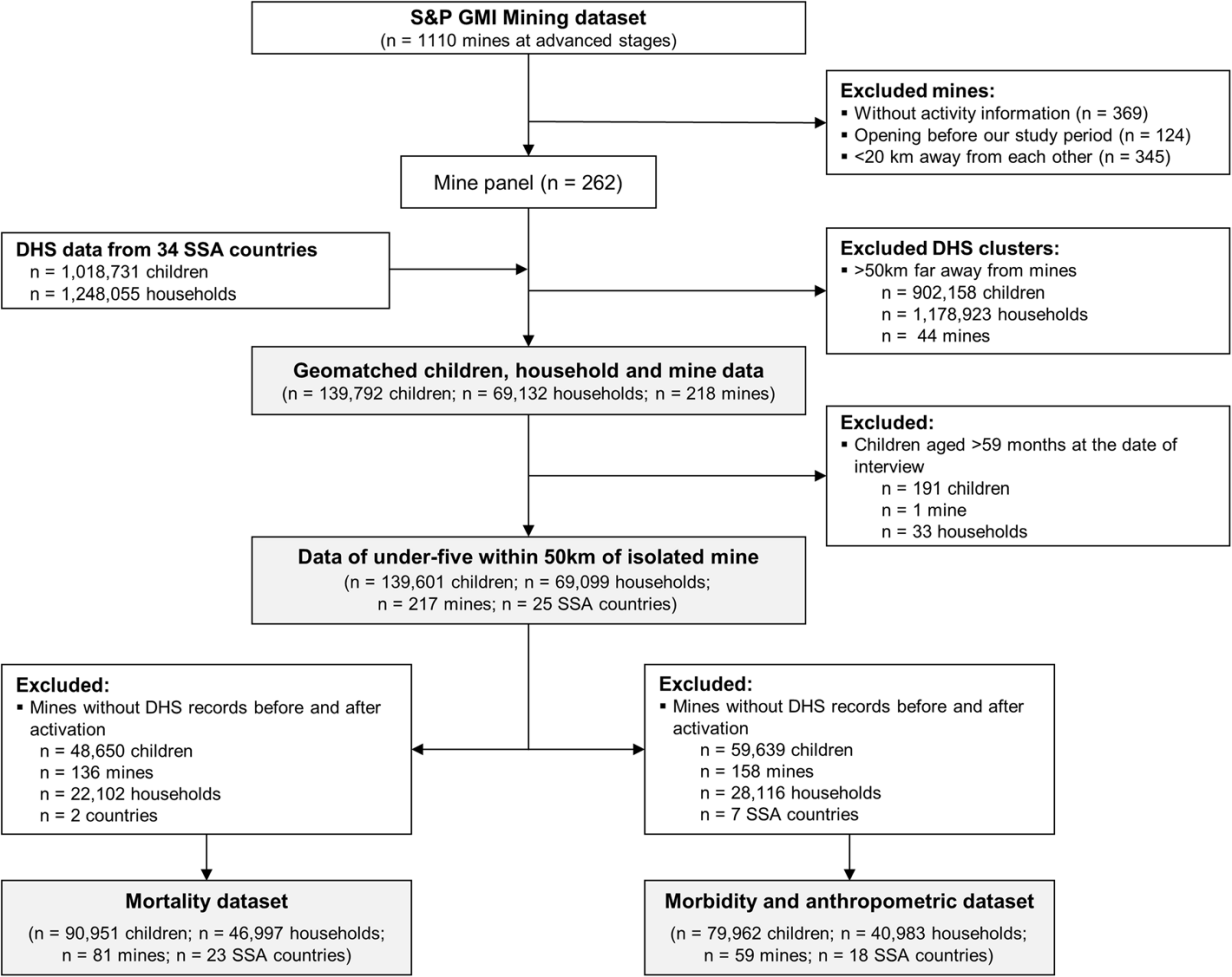
Dataset merging strategy. Note: children can occur in multiple comparisons. See a description of the matching process below

### Study design

This is a quasi-experimental difference-in-difference (DiD) study comparing child health outcomes in areas directly surrounding mines to more distant locations from the same regions before and after mine activation [32, 33]. The primary parameter of interest is the interaction term between the DHS cluster’s proximity to a mine and the post, i.e., observations made after the mine was activated. The interaction term estimates the additional change (improvement) in health outcomes seen in areas close to the mines relative to other areas nearby but outside of the direct influence of the mines. The resulting estimates can be given causal interpretation as long as the common trend assumption holds; i.e., as long as the treatment (within 10 km) and control areas (10–50 km from mines) would have experienced the same changes in health outcomes in the absence of the mining project.

### Selected variables

#### Outcome variables

In the present study, our centred attention is three primary child health outcomes. Firstly, we analysed child mortality indicators. All DHS surveys record all children born to the mothers in the last five years and the time point of any child death. Based on the information for age-at-death included in the DHS data, we computed a dummy variable indicating age-specific survival status (i.e. died or alive) for neonates (0–30 days), post-neonates (1–11 months), and children (12–60 months). While we kept the original DHS definition for under-five and child mortality [34], we computed neonatal and post-neonatal mortalities as children who died before reaching the age of 1 and 12 months, respectively. To calculate post-neonatal and child mortality rates, we only included children that had survived the first month or the first year, respectively. Missing data for children’s age at death was imputed using a hot deck approach by taking the same age at death as the last child encountered the same birth order in the data file [35].

Secondly, we analysed child morbidity indicators. The DHS datasets include morbidity data for all children under-5 years living at the survey time. We used information on whether a child experienced diarrhoeal or cough episodes in the last two weeks before the survey date. Of note, “don’t know” responses were recoded into “missing values”.

Thirdly, we analysed child anthropometrics data: to compute the z-scores of height-for-age, weight-for-height, and weight-for-age, DHS surveys collect data on height (in centimetres) and weight (in kilograms) for all living children aged under-five years in the household and the age of the child in months. Height-for-Age (HAZ), Weight-for-Age (WAZ), and Weight-for-Height (WHZ) z-scores were then calculated using standardised reference growth curves [35].

#### Exposure variables

The primary exposure variable in our analyses was the interaction of the distance to the mine (impacted and comparison clusters) and the mine’s activity status at the year of childbirth (for child mortality) and the year of DHS survey (for morbidity and anthropometric indicators). Two variable definitions were used to determine the mine’s activity status. For the primary analyses, mine activation (including the planning, exploration, prospection, and construction activities) was assumed to be at 10 years before the launch year (year zero) of mineral extraction (from now on referred to as “extraction onset”). Therefore, children born or surveyed less than 10 years before the extraction onset or later were considered exposed to an active mine, while children born/surveyed before were used as the reference group. The active mining phase was further divided into four phases corresponding to 5-year intervals for secondary analysis. These phases were defined relative to the year of extraction onset, namely: (i) the planning phase – 9 to 5 years before the extraction onset, (ii) prospection and construction phase – 4 years to extraction onset year, (iii) early extraction phase - between 1 to 5 years after the extraction onset and (iv) advanced extraction phase - more than 5 years after the extraction onset. The last phase was summarised in one category due to the low sample size. As for the dichotomous temporal categories, the time before mining activation (i.e., 10 years or more before the extraction onset) was used as the reference group.

#### Covariates

Many covariates were included in the analysis to adjust for child, maternal, and household characteristics. Child-level covariates included sex, age in completed months, twin birth, and a child’s birth order. Child age and birth order variables were recoded into 5 and 6 categories. At the maternal level, the included covariates were the highest education level, maternal age in five year-groups, and the total number of children born to women. We merged the “higher education” with “secondary education” responses and dichotomised the number of children at a cut-off value of five and above. Lastly, the household characteristics included were wealth index quintile and household location (i.e., rural vs urban). Beyond covariates, we included the mine fixed effect term in all models to account for spatial (i.e., mine location) and year fixed effect to account for temporal (i.e., year of the survey and year of childbirth) variability.

### Statistical analysis

The descriptive statistics for child health outcomes and covariate variables were double stratified by mine activation status and the distance between the DHS cluster and the mine. Logistic maximum likelihood models for binary outcomes variables (i.e., mortality, diarrhoeal, and cough episodes) and ordinary least-squares linear regression models for continuous outcome variables (i.e., anthropometric z-scores) were estimated. The regressions control for child-, maternal- and household-level factors. In addition, mine and year (childbirth year for mortality outcomes and survey year for morbidity and anthropometric outcomes) are included as fixed effects, respectively.

We assume that there are similar trends in the outcome variables across years in the absence of a causal effect induced by the presence of the mine activation [6, 7, 14, 33] and that the location of the mine projects and their activity status are not systematically correlated with other factors affecting our main outcome variables [33]. We tested this assumption by plotting child health outcomes stratified by DHS cluster’s proximity to the mine and mine activity status against the mine life stages periods.

#### Main specification

In the main analysis, we investigated the child health impact of mine activation using the interaction between the clusters’ distance to the mine and the dichotomous mine’s activity status at the year of childbirth for mortality analysis and the DHS survey year for morbidity and anthropomentric analysis (i.e., active vs non-active mine). This approach allowed us to compare the change in the prevalence of child health outcomes between the treatment group (interaction term takes the value one) and the control group (interaction term takes the value zero).

#### Alternative specification

For the secondary analysis, an alternative specification was used to investigate child health impact throughout the mine life stages (time-varying effects of mine exposure). For this purpose, the interaction term between the clusters’ distance to the mine and the four-phased mine’s activity status (planning, prospection and construction, early extraction, and advanced extraction phases) was used. In this approach, the prevalence of child health outcomes of each treatment group (interaction terms take values between 1 and 4) is compared against a unique control group (interaction term takes the value zero).

#### Sensitivity analysis

Given that mines may affect populations beyond the predefined 10 km boundary, we explore alternative exposure definitions in our sensitivity analysis. Specifically, we exclude these areas from the analyses by introducing an increasingly large buffer of potentially affected areas (i.e. 10–15 km, 10–20 km, and 10–25 km) around our treatment areas. This should also reduce misclassification concerns related to up to 5 km random noise added to DHS cluster coordinates.

The regression models were estimated using the statistical software STATA version 14.2 (Stata Corporation, LLC, College Station, TX, USA). Statistics are reported as Odds Ratio (OR; logistic regression) and beta coefficients (linear regression) where applicable, with 95% Confidence Intervals (95% CI) clustered at the survey-cluster level. *P*-values lower than 0.05 were considered significant.

## Results

### Descriptive statistics

Two separate datasets were constructed and used in the study: (i) a data set focusing on child mortality and (ii) a data set containing all available information on childhood morbidity and anthropometric datasets (Fig. 2). Below, the descriptive statistic of the childhood mortality dataset is outlined, while the descriptive statistic of the childhood morbidities and anthropometrics is given in the Additional File 1.

The final child mortality dataset contains a subset of data from 72 cross-sectional DHS datasets from 23 out of 34 SSA countries (67.6% coverage) (see Table 1). Ninety thousand nine hundred fifty-one children from 46,997 households around 81 mining projects were included. The Additional File 2 shows the complete list of included mines, country location, the year of extraction onset, the primary extracted commodities, and the total number of observations before and after mine activation. Some clusters with few observations were included in some countries, such as Gabon and South Africa, most probably located near a mine in a neighbouring country.

**Table 1:** Dataset composition, including country name, country code, survey years, and total observations per country

Country name and DHS code	Survey years	Observations	Percentage
Angola (AO)	2007, 2016	173	0.19
Burkina Faso (BF)	1993, 1998, 1999, 2003, 2010, 2014, 2017, 2018	17,885	19.66
Burundi (BU)	2010, 2011, 2012, 2013, 2016, 2017	7902	8.69
Congo Democratic Republic (CD)	2007, 2013, 2014	448	0.49
Ivory Coast (CI)	1994, 1998, 1999, 2012	1347	1.48
Gabon (GA)	2012	333	0.37
Ghana (GH)	1993, 1994, 1998, 1999, 2003, 2008, 2014, 2016	5272	5.80
Guinea (GN)	1999, 2005, 2012, 2018	5074	5.58
Kenya (KE)	2003, 2008, 2009, 2014, 2015	2767	3.04
Liberia (LB)	2006, 2007, 2008, 2009, 2011, 2013, 2016	4476	4.92
Lesotho (LS)	2004, 2009, 2014	29	0.03
Madagascar (MD)	1997, 2008, 2009, 2011, 2013, 2016	1150	1.26
Mali (ML)	1995, 1996, 2001, 2006, 2012, 2013, 2015, 2018	14,343	15.77
Mozambique (MZ)	2011, 2015, 2018	905	1.00
Nigeria (NG)	2003, 2008, 2013, 2015, 2018	946	1.04
Niger (NI)	1992, 1998	71	0.08
Namibia (NM)	2000, 2006, 2007, 2013	172	0.19
Sierra Leone (SL)	2008, 2013, 2016	3402	3.74
Senegal (SN)	1993, 1997, 2005, 2008, 2009, 2010, 2011, 2012, 2013, 2014, 2015, 2016	11,918	13.10
Tanzania (TZ)	1999, 2007, 2008, 2010, 2011, 2012, 2015, 2016, 2017	3132	3.44
South Africa (ZA)	2016	5	0.01
Zambia (ZM)	2016, 2016, 2016, 2016	1538	1.69
Zimbabwe (ZW)	1999, 2005, 2006, 2010, 2011, 2015	7663	8.43
Total		90,951	100.00

The main descriptive statistics for child, mother and household-level characteristics are presented in Table 2. Most children (70.1%; *N* = 63,790) were born after mine activation. Table 2 also shows some differences among comparison and impacted groups in pre and post-mine activation periods. Overall, 3.4% (*N* = 3095) of children were born close to active mines. Child mortality was similar in impacted and comparison areas before mine activation and improved over time (was on average, lower after mines opened).

**Table 2:** Descriptive statistics for selected maternal and child factors

Variables	Total *n* = 90,951	Birth before mine activation (***n*** = 27,161)		Birth after mine activation (n = 63,790)	
		Impacted [0–10 km]	Comparison [10–50 km]	Impacted [0–10 km]	Comparison [10–50 km]
		*n* = 1073	*n* = 26,088	*n* = 3095	*n* = 60,695
**Child mortality indicators**					
Child death (0–59 months)	6995 (7.7%)	125 (11.7%)	2848 (10.9%)	166 (5.4%)	3856 (6.4%)
Neonatal death (0–30 days)	2699 (3.0%)	58 (5.4%)	977 (3.8%)	66 (2.1%)	1598 (2.6%)
Post-neonatal death (1–11 months)	2314 (2.6%)	31 (3.1%)	963 (3.8%)	57 (1.9%)	1263 (2.1%)
Child death (12–59 months)	1982 (2.3%)	36 (3.7%)	908 (3.8%)	43 (1.5%)	995 (1.7%)
**Child characteristics**					
Child is male	46,149 (50.7%)	521 (48.6%)	13,164 (50.5%)	1556 (50.3%)	30,908 (50.9%)
Child is single birth	87,585 (96.3%)	1031 (96.1%)	25,099 (96.2%)	2994 (96.7%)	58,461 (96.3%)
Child age (months): n (mean; sd)^¥^	83,956 (28.2; 17.3)	948 (31.2; 17.9)	23,240 (30.7; 17.4)	2929 (27.4; 17.2)	56,839 (27.2; 17.1)
Birth order of a child: n (mean; sd)	90,951 (3.5; 2.4)	1073 (3.9; 2.5)	26,088 (3.9; 2.6)	3095 (3.3; 2.2)	60,695 (3.4; 2.3)
**Maternal characteristics**					
Mother’s age (years): n (mean; sd)	90,951 (29.0; 7.0)	1073 (29.1; 7.3)	26,088 (29.2; 7.1)	3095 (28.7; 6.8)	60,695 (28.9; 7.0)
Mother no education	52,274 (57.5%)	738 (68.8%)	17,722 (67.9%)	1498 (48.4%)	32,316 (53.3%)
Mother primary education	21,180 (23.3%)	184 (17.2%)	4770 (18.3%)	773 (25.0%)	15,453 (25.5%)
Mother secondary and higher education	17,492 (19.2%)	151 (14.1%)	3596 (13.8%)	824 (26.6%)	12,921 (21.3%)
Mother born less than 5 children	57,898 (63.7%)	653 (60.9%)	15,634 (59.9%)	2135 (69.0%)	39,476 (65.0%)
**Household (HH) characteristics**					
HH wealth: poorest quintile	17,452 (19.4%)	272 (25.4%)	4643 (18.4%)	529 (17.1%)	12,008 (19.8%)
HH wealth: second poorest quintile	19,600 (21.8%)	224 (20.9%)	5313 (21.1%)	684 (22.1%)	13,379 (22.0%)
HH wealth: third quintile	18,041 (20.0%)	167 (15.6%)	5039 (20.0%)	655 (21.2%)	12,180 (20.1%)
HH wealth: fourth quintile	17,011 (18.9%)	256 (23.9%)	4904 (19.5%)	708 (22.9%)	11,143 (18.4%)
HH wealth: fifth quintile (richest)	17,957 (19.9%)	154 (14.4%)	5299 (21.0%)	519 (16.8%)	11,985 (19.8%)
HH location is rural	62,644 (68.9%)	869 (81.0%)	18,014 (69.1%)	2157 (69.7%)	41,604 (68.6%)

¥ Live children only; sd – standard deviation

Descriptive statistics are stratified by time to mine activation (i.e., ten years before the extraction) and the DHS clusters’ distance to the mining sites. Data from 72 Demographic and Health Surveys from 23 SSA countries. The included DHS data was collected between 1992 and 2018 and restricted to clusters within 50 km from isolated mines (i.e., mines separated at a minimum distance of 20 km from each other). All measures represent unweighted sample proportions

### Child mortality

**Fig. 3:**
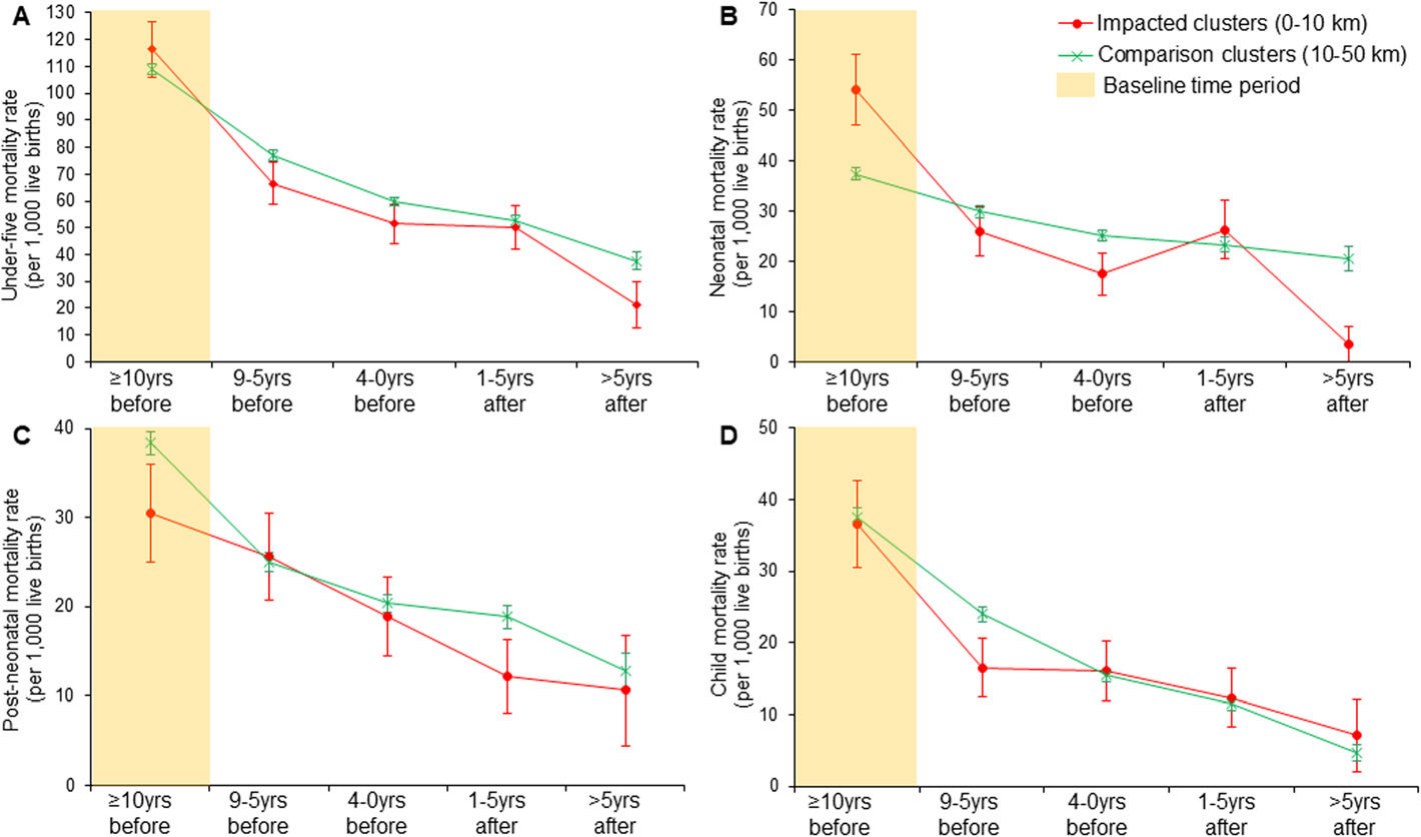
Time and spatial trend on crude mortality rate. Under-five mortality rate (panel A) and crude age-specific neonatal (panel B), post-neonatal (panel C), and child (panel D) mortality rates. The time corresponds to years relative to extraction onset (x-axis). The yellow shade illustrates the baseline period used in the regression models. Error bars show standard errors clustered at the survey-cluster level

The time and spatial trends of under-five and age-specific crude mortality rates (deaths/1000 live births) in impacted and comparison groups are illustrated in Fig. 3. Similar mortality rates before mine activation are seen for under-five (panel A) and child mortality (panel D). An overall positive impact of mine activation is observed for all mortality indicators. Indeed, a noteworthy drop in the crude under-five mortality rate is observed during the advanced extraction phase in areas close to active mines (see panel A, Fig. 3). The same effect is observed during the prospection and construction phase and the advanced extraction phase for crude neonatal mortality (see panel B, Fig. 3). A similar trend is observed for older children (Fig. 3, panels C and D), except for an observed considerable decline during the planning and early extraction phases for child and post-neonatal crude mortality rate, respectively.

Table 3 shows the average impact on child mortality indicators. On average, mine activation is associated with lower odds for neonatal mortality rate (aOR: 0.55, 95% CI: 0.37–0.83; column (2)). No statistically significant changes were observed for under-five (column (1)), post-neonatal (column (3)), and child (column (4)) mortality overall.

**Table 3:** Estimates of association between mine exposure and child mortality indicators using the main specifications

Interaction (proximity*active)$	(1)	(2)	(3)	(4)
	Under-five mortality (0–59 months)	Neonatal mortality (0–30 days)	Post-neonatal mortality (1–11 months)	Child mortality (12–59 months)
Crude model**†**	0.78	0.55**	1.11	0.86
	(0.58–1.04)	(0.36–0.83)	(0.69–1.80)	(0.52–1.43)
Observations	90,951	90,951	88,252	85,938
Adjusted model**‡**	0.88	0.55**	1.22	1.17
	(0.68–1.14)	(0.37–0.83)	(0.81–1.86)	(0.75–1.82)
Observations	90,056	89,812	87,281	82,558

* p < 0.05, ** p < 0.01

**$** - interaction term between clusters’ proximity (0–10 km) and the mine activity status at childbirth year; † − model including interaction term only; **‡** − model adjusted for gender, twin births, birth order, number of children ever born to mother, maternal age, maternal education, residence, wealth index, mine, and birth year

The treatment group corresponds to children born within 10 km from active mines. The reference group (control) are children born within a distance radius of 10 km before mine activation and those born 10–50 km away regardless of mine activity status

The estimates are relative to the year of childbirth using logistic regression models. The reported estimates are crude and adjusted odds ratio (OR), and the 95% confidence intervals (CIs) are shown in parentheses and are clustered at the survey-cluster level

Table 4 shows estimated impacts stratified by time relative to the start of mine extraction, i.e., the year of extraction onset. For neonatal mortality, we find largest reductions during the prospection and construction phase (aOR: 0.43, 95% CI: 0.25–0.75), as well as in the advanced extraction phase (aOR: 0.10, 95% CI: 0.02–0.61; column (2)). Post-neonatal and child mortality appear to increase slightly, but the rise is not statistically significant.

**Table 4:** Estimates of association between child mortality indicators and the interaction of mining proximity (0–10 km vs 10–50 km) and the mine life stages using alternative specifications

Interaction (proximity*active)$	(1)	(2)	(3)	(4)
	Under-five mortality (0–59 months)	Neonatal mortality (0–30 days)	Post-neonatal mortality (1–11 months)	Child mortality (12–59 months)
Close* planning phase (9–5 years before)	0.93	0.62	1.50	0.90
	(0.67–1.27)	(0.36–1.04)	(0.89–2.54)	(0.49–1.65)
Close*prospection and construction phase (4–0 years before)	0.84	0.43**	1.21	1.40
	(0.60–1.19)	(0.25–0.75)	(0.68–2.13)	(0.81–2.41)
Close*early extraction phase (1–5 years after)	1.04	0.81	0.87	1.67
	(0.68–1.58)	(0.46–1.43)	(0.43–1.78)	(0.68–4.12)
Close*advanced extraction phase (> 5 years after)	0.43	0.10*	0.89	1.17
	(0.16–1.18)	(0.02–0.61)	(0.30–2.68)	(0.30–4.51)
Observations	90,056	89,812	87,281	82,558

* p < 0.05, ** p < 0.01

**$** - interaction term between clusters’ proximity (0–10 km) and the mine activity status at childbirth year; All models are adjusted for child sex, twin births, maternal age, maternal education, residence, wealth index, birth order, number of children ever born to mother, mine, and birth year

The treatment group corresponds to children born within a distance radius of 10 km from active mines, categorised in four mine life stages. The reference group (control) are children born within 10 km before mine activation plus those born 10–50 km away regardless of mines’ activity status

Mine life stages stratify all logistic regression estimations compared against the reference comprised of the interaction between clusters located at 10–50 km and all periods of mine life stages

The reported estimates are crude and adjusted odds ratio (OR), and the 95% confidence intervals (CIs) are shown in parentheses and are clustered at the survey-cluster level

### Child morbidity

**Fig. 4:**
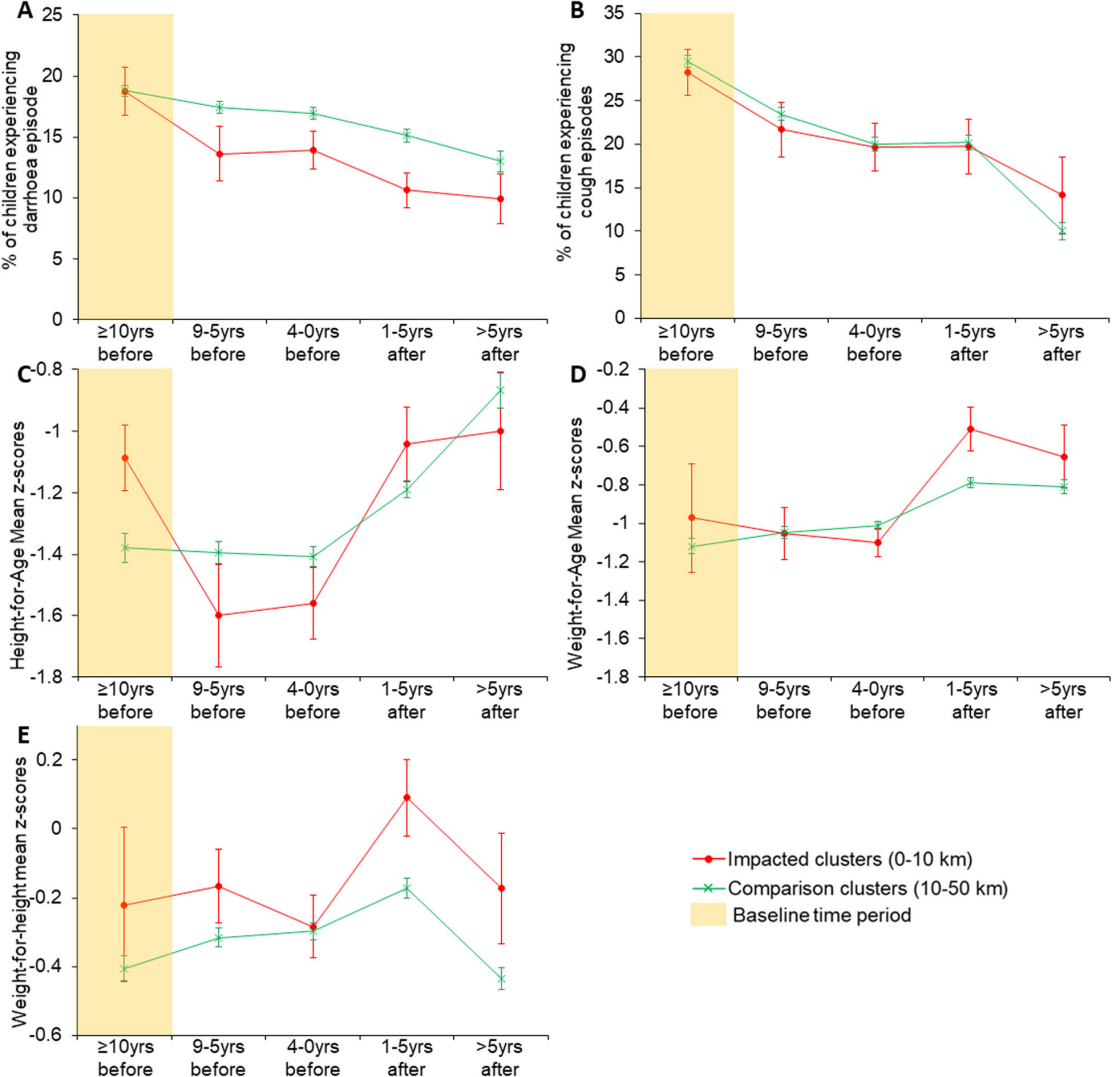
Morbidity and child anthropometrics trends in impacted and comparison areas. Panel A, diarrhoea; panel B, cough; panel C, height-for-age z-scores; panel D weight-for-age z-scores and panel D, weight-for-age z-scores. Temporal comparison is relative to the mine extraction period (x-axis), and spatial comparison is relative to the cluster’s proximity to the mine (impacted, 0–10 km vs comparison, 10–50 km areas). The yellow shade illustrates the baseline period used in the regression models. Error bars show standard errors clustered at the survey-cluster level

Figure 4 shows the relative change in diarrhoea (panel A) and cough (panel B) prevalence over time (years before and after mine activation) and cluster proximity (0–10 km and 10–50 km) at the year of the DHS survey. Overall, diarrhoea prevalence in impacted areas declined considerably after mine activation compared with diarrhoea cases in comparison areas. Although similar in both areas over mine stages, a decline is also observed for cough prevalence.

The impacts of the mine activity status on the anthropometrics mean z-scores are shown in panels C-E of Fig. 4. The trend of the mean HAZ is shown in panel C and ranges from − 1.6 and − 0.9 standard deviations (SDs) over the study period. When compared to children in comparison areas (i.e., 10–50 km away), mine activation seems to trigger a rapid decrease in mean HAZ, followed by an increase, but similar trends between the two groups.. The mean WAZ decreases after mine activation among children living nearby (0–10 km) but remains similar to those living far from active mines. Of note, the mean WAZ increases substantially soon after the extraction onset.. Moreover, the mean WAZ is above − 1.2, but below − 0.4 SDs, an indication of a low probability of underweight and overweight children over the study period (Fig. 4, panel D). The anthropometric WHZ seems to be much more positively affected than HAZ and WAZ measures (see Fig. 4, panel E), ranging from − 0.5 to 0.2 SDs over the study period. Again, mine activation seems to trigger an increase in the mean WHZ. It is important to note that the mean WHZ among children living close to an active mine remains higher over the study periods than children in comparison areas, nevertheless similar between the period before mine activation and in the advanced extraction phase. The mean WHZ is notably increased soon after the extraction onset.

Table 5 shows the logistic regression results for diarrhoeal and cough episodes (columns (1) and (2)) and the linear predictions for anthropometrics z-scores (i.e., HAZ, WAZ and WHZ) (columns (3–5)). We found an indication of significant protection for experiencing diarrhoea among children living near active mines. The risk for diarrhoea significantly decreases by 32% soon after mine activation (aOR: 0.68, 95% CI: 0.51–0.90) (column (1)). Although not significant, children living in mining areas seems to experience a decreased percentage points on their mean HAZ (column (3)) but increased WAZ and WHZ z-scores (columns (4 and (5)).

**Table 5:** Estimates of association between child health outcomes, anthropometrics, and mining exposure using the main specifications

Interaction (proximity*active)$	(1)	(2)	(3)	(4)	(5)
	Diarrhoeal episodes	Cough episodes	Height-for-Age z-scores	Weight-for-Age z-scores	Weight-for-Height z-scores
Crude model^**†**^	0.74	1.01	−0.35*	−0.07	− 0.03
	(0.54–1.01)	(0.73–1.42)	(−0.62 - -0.08)	(− 0.64–0.50)	(−0.50–0.43)
Observations	59,868	58,593	35,027	35,609	34,594
Adjusted model^**‡**^	0.68**	1.01	−0.16	0.10	0.10
	(0.51–0.90)	(0.77–1.31)	(−0.40–0.08)	(−0.28–0.48)	(−0.28–0.48)
Observations	59,078	57,799	35,027	35,609	34,594

* p < 0.05, ** *p* < 0.01

**$** - interaction term between clusters’ proximity (0–10 km) and mine activity status at survey year; † − model including interaction term only; **‡** − adjusted for gender, child age, twin births, maternal age, maternal education, residence, wealth index, birth order, number of children ever born to mother

The treatment group corresponds to children located within a distance radius of 10 km from active mines at the DHS survey year. The reference group (control) are children located within a distance radius of 10 km before mine activation and those born 10–50 km away regardless of mines’ activity status at the DHS survey year

Logistic regression models are used for estimating the odds ratio for diarrhoeal, and cough episodes (columns (1) and (2)) and linear regression models are used for anthropometric indicators (columns (3), (4), and (5)). The reported estimates for morbidities (i.e., diarrhoea and cough) are crude and adjusted odds ratios (OR), and the child’s anthropometrics are crude and adjusted beta coefficients. The 95% confidence intervals (CIs) are shown in parentheses and are clustered at the survey-cluster level

The time-specific variation’s results for the interaction term between mine proximity to the survey cluster and the period of mine activity are illustrated in Table 6. We investigate the effect in four periods of 5-years each in the mine life stages. While the risk for diarrhoea decreases over time, the significant effect of the interaction on the risk for a child experiencing diarrhoea episodes is seen during the prospection and construction phase (aOR: 0.69, 95% CI: 0.49–0.97) (see Table 6, column (1)). Conversely, the risk for a child to experience cough episodes among those living close to an active mine is seen to increase over the study period, particularly during the advanced extraction phase, although not statistically significant (aOR: 1.51, 95% CI: 0.80–2.86).

**Table 6:** Estimates of association between child health outcomes, anthropometrics, and the interaction of mining proximity (0–10 km vs 10–50 km) and the mine life stages using alternative specifications

Interaction (proximity*mining phase)$	(1)	(2)	(3)	(4)	(5)
	Diarrhoeal episodes	Cough episodes	Height-for-Age z-scores	Weight-for-Age z-scores	Weight-for-Height z-scores
Close* planning phase (9–5 years before)	0.73	1.05	−0.20	0.03	0.03
	(0.48–1.10)	(0.74–1.48)	(− 0.53–0.14)	(−0.39–0.45)	(−0.42–0.47)
Close*prospection and construction phase (4–0 years before)	0.69*	0.96	−0.28*	− 0.00	0.06
	(0.49–0.97)	(0.67–1.39)	(−0.55 - -0.01)	(− 0.39–0.39)	(−0.33–0.46)
Close*early extraction phase (1–5 years after)	0.69	1.06	−0.04	0.22	0.17
	(0.47–1.02)	(0.70–1.61)	(− 0.34–0.26)	(−0.19–0.62)	(−0.23–0.57)
Close*advanced extraction phase (> 5 years after)	0.55	1.51	0.03	0.36	0.25
	(0.26–1.17)	(0.80–2.86)	(− 0.40–0.46)	(−0.09–0.82)	(−0.16–0.66)
Observations	59,078	57,799	35,027	35,609	34,594

* *p* < 0.05, ** p < 0.01

**$** - interaction term between clusters’ proximity (0–10 km) and the mine activity status at survey year

All models are adjusted for child sex, twin births, maternal age, maternal education, residence, wealth index, birth order, number of children born to mother, mine and birth year

The treatment group corresponds to children located within a distance radius of 10 km from active mines at the DHS survey year, categorised in four mine life stages. The reference group (control) are children located within a distance radius of 10 km before mine activation plus those born 10–50 km away regardless of mines’ activity status at the DHS survey year

Mine life stages stratify all regression estimations compared against the reference comprised of the interaction between clusters located at 10–50 km and all periods of mine life stages

Logistic regression models are used for estimating the odds ratio for diarrhoeal, and cough episodes (columns (1) and (2)) and linear regression models are used for anthropometric indicators (columns (3), (4), and (5)). The reported estimates for morbidities (i.e., diarrhoea and cough) are crude and adjusted odds ratios (OR), and the child’s anthropometrics are crude and adjusted beta coefficients. The 95% confidence intervals (CIs) are shown in parentheses and are clustered at the survey-cluster level

While the effect of the interaction on children’s nutritional indicators over time does not show a clear pattern, we found a significant decrease of 28 percentage points on the mean HAZ during the prospection and construction phase, an indication of an increased rate of children shorter for their age in this mining phase across mining areas (Table 6, columns (3)). It is worth noting that the percentage points of the mean z-score of weight-for-age and weight-of-height increase over the study period, although all statistically insignificant (Table 6, columns (4) and (5)).

### Sensitivity analysis

**Fig. 5:**
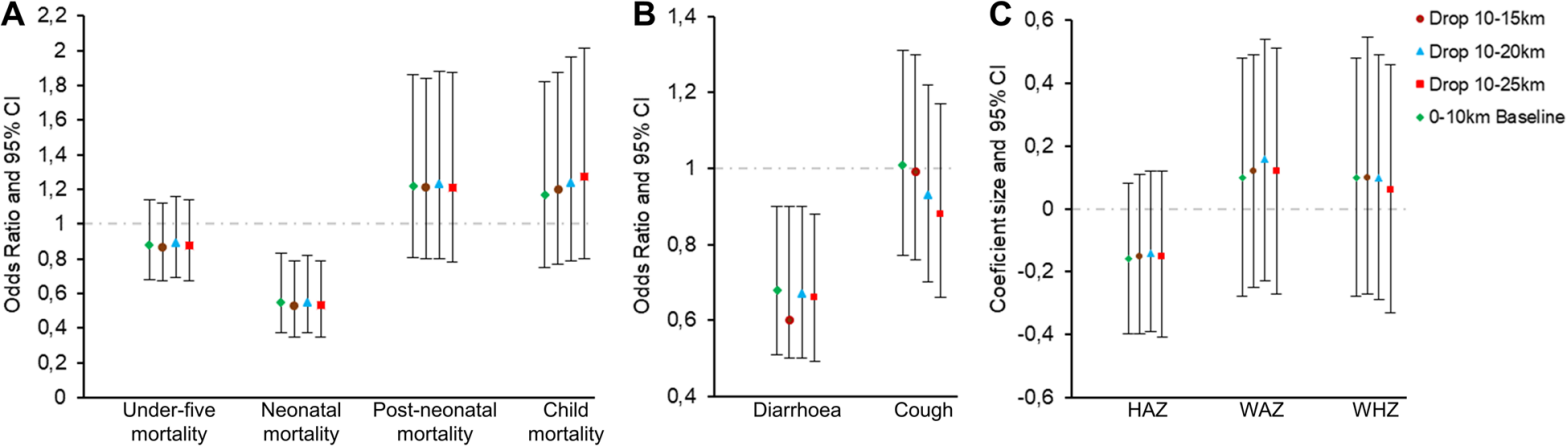
Sensitivity analysis of all child health indicators using logistic (mortality and morbidities) and linear (anthropometrics) regression models. Estimates are adjusted Odds Ratios of under-five and age-specific mortality rates (panel A) and child morbidities (panel B) and adjusted beta coefficients of child anthropometrics (panel C). The baseline specification model (control group is the entire 10–50 km area) is included for comparison. Error bars show 95% confidence intervals clustered at the survey-cluster level. bef - before; yrs. - years

Results of the regression model sensitivity analysis are presented in Fig. 5. In all comparisons, the first bar (green diamond) represents the baseline point estimates from Table 3 and Table 5. The remaining bars show results when excluding 10–15 km (red dot), 10–20 km (blue triangle) and 10–25 km (red square) areas. We do not observe significant changes in the estimated impacts on either outcome.

## Discussion

This paper assessed the impact of 81 large-scale mining projects launched in 23 SSA countries between 2002 and 2019. We found that mine activation lowered the risk for neonatal mortality by 45% and the risk for childhood diarrhoea by 32% among children born and living within 10 km of an active mine compared to children living further away. However, no immediate impact on cough and nutritional status was seen. Looking more closely at the timing of observed changes in health outcomes, we observed that the risk for neonatal mortality reduced significantly during the early mining phases (by 53% in the prospection and construction and 90% during the advanced extraction phases). The odds for childhood diarrhoea decreased by 31% and the mean height-for-age z-scores reduced by 28 percentage points during the prospection and construction phase.

The reduced neonatal mortality close to mines is in line with other studies using DHS data [4, 7], which have documented similar decreases in infant mortality in the first 6 and 12 months of children’s life [4, 7]. The present study showed the primary benefits during the neonatal period, while no impacts on post-neonatal mortality were found. Another study investigating infant mortality around gold mines across SSA reported mixed effects, with impact heterogeneity primarily driven by mine location [6]. Our analysis shows that the lack of effect on post-neonatal mortality may come from the large and almost instant drops in mortality rates experienced by communities far away from mines, while no significant decline in the vicinity of the mines at the time of mine activation is observed. Drops in the risk for neonatal mortality around recently opened mines are often linked with increases in local welfare and women empowerment [4, 7]. Studies have reported that women living close to mines are more likely to have formal education, have better jobs, earn more income and live in wealthier households [17, 31]. These changes could contribute to reducing neonatal mortality in mining areas, as observed in our study.

Our results suggest that the impact on neonatal mortality risk likely differs substantially across the mining life stages. We found mortality reductions mainly during the prospection and construction phase and the advanced extraction phases. The pre-extraction period typically corresponds to the mine investment period, which generates local employment and consequently household economic growth [6, 17, 36]. The post-extraction effects are more surprising, as mine-related pollution from extraction activities might increase over time and offset the positive employment and income effects seen in the initial stages [23]. During the latter stages of the mining projects, improvements can be explained by further economic development or health promotion activities supported by mines. In line with this hypothesis, it is argued that the size of primary exports by mines increases in later stages during the resource extraction phase [36, 37]. Consequently, they may contribute more to local and national economic growth and potentially ramp up corporate social responsibility activities, such as investments in local water and sanitation infrastructures [8, 36, 38, 39]. Overall, these results suggest that the impact of mining projects on local development [6, 40, 41] might be an opportunity for African countries to work towards the ambitious target of the 2030 Agenda for Sustainable Development to curb infant and under-five mortality [42].

Only a few studies examine the effect of mines on child morbidity and malnutrition [8, 14, 17]. Using similar data, Dietler and colleges found no effect of mining activities on diarrhoea prevalence [8]. Our findings suggest that mine activation reduces the risk for a child experiencing diarrhoeal episodes if living within 10 km. In addition, the impact of mining projects on childhood diarrhoea may be more prominent during the prospection and construction phase of the mine. Fluctuations of impacts according to the stage of mine developments have been reported by other studies [27, 28]. In our study, the differential effect size depending on the mining life stages may explain the absence of consistent findings in other studies [6, 43].

Although curable, diarrhoeal diseases remaina common cause of death for young children in SSA countries [6, 41]. Many of these deaths attributed to poor water and sanitation infrastructures [39, 44–47]. The opening of many large-scale mining projects in the last two decades represents an excellent opportunity for lowering both prevalence of and mortality due to diarrhoea [1, 2, 6]. The planning, construction, and early extraction periods are capital and investment-intensive. In addition, intense corporative social responsibility interventions on water and sanitation and job creation characterise these project phases and, thus, are more likely to decrease waterborne diseases such as diarrhoea [8, 38]. Further, this economic development is concurrent with women’s empowerment, which can facilitate investments in child health and, thus, disease prevention [4, 30, 43]. Further investigation is warranted to illustrate economic growth translation into health gain at the local level in industrial mining areas.

Contrary to diarrhoea, there was a sharp increase in the risk for cough episodes in impacted areas over the study period, particularly later in the advanced extraction phase, although not statistically significant. These results are similar to those of previous studies reporting increased likelihood for respiratory-related diseases in children living in mining communities [48, 49] and may reflect the increased levels of air pollution around active mines found in other studies [50–53]. Additionally, mining-related and other environmental pollution have been associated with poor child health outcomes, including respiratory diseases [23, 54, 55]. Our findings, however, point to a need for further research to better understand the distribution pattern across countries and different types of mines. In addition, managing air pollution around recently active mining projects could help reduce the respiratory-related disease burden among young children.

We found no evidence of the effect of mining on any nutrition indicator when using baseline specifications. However, when exploring the timing of observed changes, children ‘s growth appears more limited during the prospection and construction phase of the mine. Our findings are in line with those reported in other studies conducted in low and middle-income countries, which found that mining activities were associated with an increased rate of poor nutritional status of children born from mothers living close to large mines [17, 56]. However, these findings do not corroborate with those reported in a similar study conducted in the context of gold mines in Colombia [14]. Romero and Saavedra reported that living near active gold mines did not affect either low birth weight or stunting in newborns [14]. However, contrary to our findings, recent evidence suggests lower stunting and underweight rates in children living in mining communities [9]. Mixed-effects of mine operations on anthropometric indicators have also been reported in three SSA countries [6, 43]. These differences may partially be explained by differences in the empirical approach used across studies. We use a different temporal exposure definition (i.e., only records from less than ten years before the mine extraction phase are considered to be exposed to mine activities). Our strategy may have affected our results in two ways: [1] the selection strategy for the control group led to smaller sample size and reduced overall statistical power, and [2] more positively, by analysing only changes over time and abstracting from cross-sectional relationships between mining locations and general population and health characteristics.

The rapid change in the land use during the prospection and construction phase of mining projects, including land-grabbing by mining companies, environmental degradation, and structural shifts in income-generating activities, can lead to food insecurity and, thus, poor nutrition status, particularly for young children [16, 27, 57]. This land change and its effect is in line with our results showing an increase in the prevalence of stunted children during the prospection and construction phase. This period is usually considered the baseline period by most studies reporting improvement in the nutritional status of children living close to recently opened mining projects [8, 17, 43]. These differences can be explained by the fact that such a definition of temporal exposure can affect the estimated effect by allocating more stunted children to the control group and thus changing the estimated effect’s direction and size. Our findings point to a need for further research to assess the temporal variations in childhood nutritional status in mining areas.

This study was guided by a well-known and extensively discussed methodology [4, 6–8, 14, 17, 30] to explore mine-induced changes in child health outcomes. The main contribution to the existing scientific literature is that the modified identification strategy and alternative specifications better investigate causal effects over the mine life stages. Nevertheless, our findings have several limitations. Firstly, our temporal exposure definition reduced the sample size and, thus, the statistical power of most of the performed analyses. Specifically, our strategy resulted in smaller sample size before mine activation, which did not allow us to see trends in the prevalence of health outcomes in the absence of the mining projects.

Similarly, a small sample size was also obtained for five years and later after the onset of extraction activities, limiting our analysis of desegregated observations at this period, i.e., between 5 and 10 years and more than ten years after the onset of extraction activities. At the same time, it allocated more children with ‘positive’ health outcomes to the treated group, which may have changed the direction and size of the estimates. Secondly, we focus on large-scale mining projects; however, a substantial proportion of the health-related effects may derive from artisanal and small-scale mining activities, which are often found in proximity to industrial mining projects [25, 45]. Thirdly, we did not exclude large cities from our sample, which could introduce some bias. Many factors may play a role in child health, and substantial differences exist between city and non-city settings. We could not adjust for several factors such as population density and urbanisation. Furthermore, self-reported data such as diarrhoea and cough are prone to recall and reporting bias. Lastly, the inaccuracy of mine GPS data and the coordinate reallocation by the DHS could have introduced errors and reduced our statistical power.

## Conclusion and recommendations

The results presented in this paper suggest that the impact of mines on child health is complex and likely non-linear over time; i.e., significant effects can be found in some mine life stages but not in others. We find evidence that the launch of industrial mining projects accelerates the improvement of neonatal survival and reduction in the risk for childhood diarrhoeal in SSA countries, with significant contributions during the prospection and construction and in the advanced extraction phases. While the launch of industrial mining projects seems not to have any impact on childhood cough and nutritional status, our evidence points to an increase in stunting rate before the launch of extraction activities and increased rate of respiratory disease symptoms once extraction starts, reflecting an increase of food insecurity and environmental pollution, respectively. Therefore, health management plans with an emphasis on maintaining positive health impacts throughout the mining life stages and addressing the identified risks on respiratory and nutritional health in children are advisable.

On the other hand, the varying effects of industrial mining on child health outcomes throughout the mining life stages may reflect differential mine-related contributions to economic growth and community development over time. Further research aiming to provide more insights into the temporal effects of mine impacts and, thus, a better understanding of these complex dynamics of health impacts are recommended. These future studies should be powered by using longitudinal data to determine whether the association between these health outcomes and mining varies based on the mining setting (e.g., type of resource extracted, country location of the mine, preventative measures taken by the company). The studies should include health monitoring data that should be part of the mine’s health mitigation and monitoring plan.

## Supplementary Information

**Additional file 1 Table A1.** Descriptive statistics of childhood morbidities and anthropometrics**.**

**Additional file 2 Table A2**. Descriptive statistics of mine projects, including hosting country and primary commodity.

## Data Availability

In the present study, secondary data was used. All transformed, generated or analysed data during the study are included in this published article and the Additional Files 1 and 2. The full dataset is also available by request at the Demographic and Health Survey (https://dhsprogram.com/) and Standard & Poor’s (S&P) Global Market Intelligence Mining websites (https://www.spglobal.com/).

## Abbreviations

aOR: Adjusted Odds Ratio
CA: California
CI: Confidence Interval
DHS: Demographic and Health Survey
DiD: Difference-in-Difference
GPS: Global positioning system
HAZ: Height-for-Age z-score
HH: Household
OR: Odds Ratio
S&P GMI: Standard & Poor’s Global Market Intelligence
SD: Standard deviations
SSA: Sub-Saharan Africa
UNDP: United Nations Development Programme
USA: United State of America
WAZ: Weight-for-Age z-score
WHZ: Weight-for-Height z-score
