# Correction: Assessing the effects of mining projects on child health in sub-Saharan Africa: a multi-country analysis

**DOI:** 10.1186/s12992-022-00816-6

**Published:** 2022-02-22

**Authors:** Hermínio Cossa, Dominik Dietler, Eusébio Macete, Khátia Munguambe, Mirko S. Winkler, Günther Fink

**Affiliations:** 1 Swiss Tropical and Public Health Institute, Kreuzstrasse 2, 4123 Allschwil, Switzerland; 2 University of Basel, P.O. Box, CH-4003, Basel, Switzerland; 3 Manhiça Health Research Centre, 1929 Maputo, Mozambique; 4 National Directorate of Public Health, Ministry of Health, 264 Maputo, Mozambique; 5 Faculty of Medicine, University Eduardo Mondlane, 3453 Maputo, Mozambique

**Correction: Global Health 18, 7 (2022)**

**https://doi.org/10.1186/s12992-022-00797-6**

Following publication of the original article [1], the authors flagged that the article had published with a duplicate of Fig. 4 in place of Fig. 5.

**Fig. 5:**
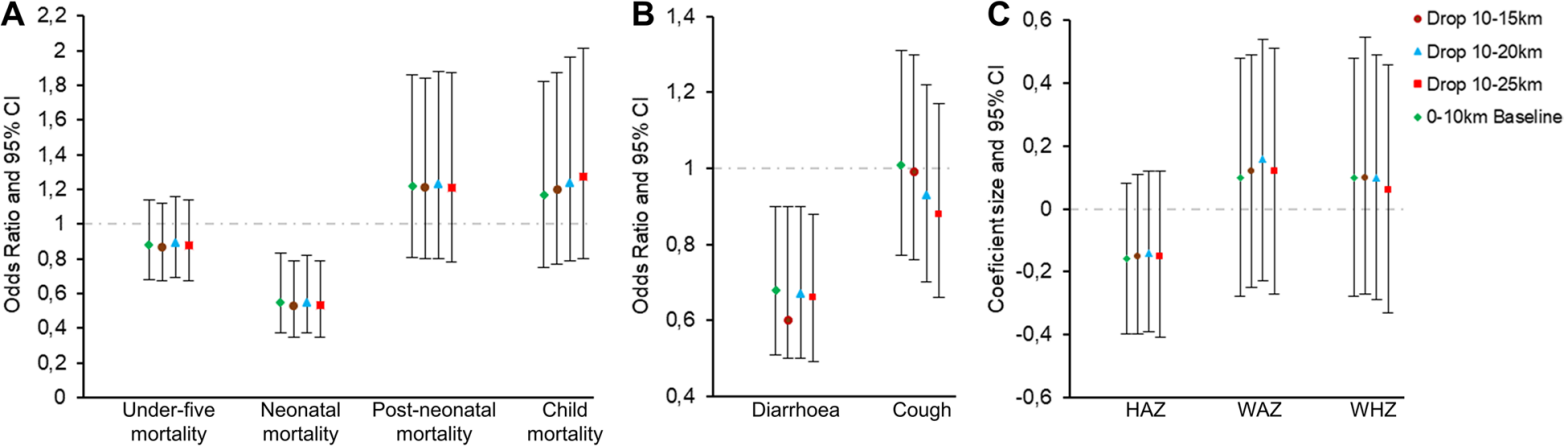
Sensitivity analysis of all child health indicators using logistic (mortality and morbidities) and linear (anthropometrics) regression models. Estimates are adjusted Odds Ratios of under-five and age-specific mortality rates (**A**) and child morbidities (**B**) and adjusted beta coefficients of child anthropometrics (**C**). The baseline specification model (control group is the entire 10–50 km area) is included for comparison. Error bars show 95% confidence intervals clustered at the survey-cluster level. bef - before; yrs. - years

Figure 5 has now been corrected in the published article and may be found in this erratum.
