# Supplementary material for: Assessing the effects of mining projects on child health in sub-Saharan Africa: a multi-country analysis
Source: Global Health. 2022 Jan 31;18:7. doi: 10.1186/s12992-022-00797-6 (PMC8802519; doi:10.1186/s12992-022-00797-6)
Supplement: Supplementary file 1 — Additional file 1 Table A1. Descriptive statistics of childhood morbidities and anthropometrics. [file 12992_2022_797_MOESM1_ESM.docx]

**Table A2.** Descriptive statistics of mine projects, including hosting country and primary commodity.

| **Mine Name** | **Operator Name** | **Open Year** | **Country** | **Primary Commodity** | **Records Before Opening** | **Records After Opening** | **Total** | **Percent** |
| --- | --- | --- | --- | --- | --- | --- | --- | --- |
| Homase | GoldStone Resources Limited | 2002 | Ghana | Gold | 95 | 1 321 | 1 416 | 1.56 |
| Murowa | RioZim Limited | 2004 | Zimbabwe | Diamonds | 1 | 421 | 422 | 0.46 |
| Samira Hill | Societe de Patrimoine des Mines du Niger SA | 2004 | Niger | Gold | 45 | 162 | 207 | 0.23 |
| Loulo | Barrick Gold Corporation | 2005 | Mali | Gold | 55 | 558 | 613 | 0.67 |
| Ahafo | Newmont Goldcorp Corporation | 2006 | Ghana | Gold | 153 | 523 | 676 | 0.74 |
| Sengwa | Sengwa Colliery (Private) Limited | 2007 | Zimbabwe | Coal | 17 | 168 | 185 | 0.20 |
| Taparko | Nord Gold SE | 2007 | Burkina Faso | Gold | 198 | 870 | 1 068 | 1.17 |
| Bonikro | BDG Capital Limited | 2008 | Cote d'Ivoire | Gold | 144 | 110 | 254 | 0.28 |
| Bouroum | Nord Gold SE | 2008 | Burkina Faso | Gold | 435 | 777 | 1 212 | 1.33 |
| Hwini-Butre | Golden Star Resources Limited | 2008 | Ghana | Gold | 151 | 445 | 596 | 0.66 |
| Kalsaka/Sega | Perseus Mining Limited | 2008 | Burkina Faso | Gold | 713 | 1 811 | 2 524 | 2.78 |
| Plant 11 | Trevali Mining Corporation | 2008 | Sierra Leone | Diamonds | 124 | 1 761 | 1 885 | 2.07 |
| QMM | Rio Tinto | 2008 | Madagascar | Ilmenite | 38 | 510 | 548 | 0.60 |
| Yaoure | Perseus Mining Limited | 2008 | Cote d'Ivoire | Gold | 276 | 239 | 515 | 0.57 |
| Youga | Avesoro Resources Inc. | 2008 | Burkina Faso | Gold | 445 | 1 534 | 1 979 | 2.18 |
| Bomboko | BDG Capital Limited | 2009 | Guinea | Diamonds | 138 | 386 | 524 | 0.58 |
| Buzwagi | Acacia Mining plc | 2009 | Tanzania | Gold | 18 | 603 | 621 | 0.68 |
| Inata | Balaji Group of Companies | 2009 | Burkina Faso | Gold | 99 | 582 | 681 | 0.75 |
| Mandala-Bouro | BDG Capital Limited | 2009 | Guinea | Diamonds | 222 | 268 | 490 | 0.54 |
| Douta Alluvial | Bassari Resources Limited | 2010 | Senegal | Gold | 5 | 752 | 757 | 0.83 |
| Essakane | IAMGOLD Corporation | 2010 | Burkina Faso | Gold | 111 | 591 | 702 | 0.77 |
| Old Nic | New Dawn Mining Corp. | 2010 | Zimbabwe | Gold | 416 | 1 225 | 1 641 | 1.80 |
| Tongon | Barrick Gold Corporation | 2010 | Cote d'Ivoire | Gold | 76 | 100 | 176 | 0.19 |
| Anjin Zimbabwe | Zimbabwe Mining Development Corp | 2011 | Zimbabwe | Diamonds | 106 | 274 | 380 | 0.42 |
| Guiro | Komet Resources Inc. | 2011 | Burkina Faso | Gold | 298 | 726 | 1 024 | 1.13 |
| Liberia Mines | ArcelorMittal | 2011 | Liberia | Iron Ore | 288 | 1 744 | 2 032 | 2.23 |
| Monastery | Thabex Ltd. | 2011 | South Africa | Diamonds | 1 | 33 | 34 | 0.04 |
| Nzema | BCM International Limited | 2011 | Ghana | Gold | 141 | 178 | 319 | 0.35 |
| Tienfala | Sahara Mining | 2011 | Mali | Iron Ore | 2 662 | 6 729 | 9 391 | 10.33 |
| Ambatovy | Sherritt International Corporation | 2012 | Madagascar | Nickel | 81 | 330 | 411 | 0.45 |
| Ansongo | Transatlantic Mining Corporation | 2012 | Mali | Manganese | 201 | 289 | 490 | 0.54 |
| Bagoe River | PG Alluvial Mining Plc | 2012 | Mali | Gold | 364 | 909 | 1 273 | 1.40 |
| Forecariah | China International Fund Management Co. Ltd. | 2012 | Guinea | Iron Ore | 146 | 1 703 | 1 849 | 2.03 |
| Kibali | Barrick Gold Corporation | 2012 | Dem. Rep. Congo | Gold | 3 | 50 | 53 | 0.06 |
| Kilimapesa | Goldplat Plc | 2012 | Kenya | Gold | 243 | 1 645 | 1 888 | 2.08 |
| Kodieran | Wassoul' Or SA | 2012 | Mali | Gold | 171 | 204 | 375 | 0.41 |
| Lubambe | EMR Capital Pty. Ltd. | 2012 | Zambia | Copper | 10 | 456 | 466 | 0.51 |
| Luremo | Luminas-Sociedade Mineira de Luremo | 2012 | Angola | Diamonds | 5 | 168 | 173 | 0.19 |
| New Luika | Shanta Gold Limited | 2012 | Tanzania | Gold | 16 | 161 | 177 | 0.19 |
| Trekkopje | Orano SA | 2012 | Namibia | U3O8 | 39 | 17 | 56 | 0.06 |
| Vele | MC Mining Limited | 2012 | South Africa | Coal | 75 | 153 | 228 | 0.25 |
| Agbaou | Endeavour Mining Corporation | 2013 | Cote d'Ivoire | Gold | 157 | 123 | 280 | 0.31 |
| Akyem | Newmont Goldcorp Corporation | 2013 | Ghana | Gold | 361 | 368 | 729 | 0.80 |
| Graphmada | Bass Metals Limited | 2013 | Madagascar | Graphite | 28 | 163 | 191 | 0.21 |
| Hahotoe-Kpogame-Kpeme | Komet Resources Inc. | 2013 | Ghana | Phosphate | 197 | 298 | 495 | 0.54 |
| Kasempa | H and S Mining Ltd | 2013 | Zambia | Copper | 7 | 62 | 69 | 0.08 |
| Kwale | Base Resources Limited | 2013 | Kenya | Ilmenite | 335 | 1 065 | 1 400 | 1.54 |
| Perkoa | Trevali Mining Corporation | 2013 | Burkina Faso | Zinc | 1 346 | 1 078 | 2 424 | 2.67 |
| Sega | Perseus Mining Limited | 2013 | Burkina Faso | Gold | 1 181 | 1 311 | 2 492 | 2.74 |
| Arcturus | TN Securities (Pvt) Limited | 2014 | Zimbabwe | Gold | 1 057 | 1 829 | 2 886 | 3.17 |
| Grande Cote | TiZir Limited | 2014 | Senegal | Ilmenite | 876 | 1 582 | 2 458 | 2.70 |
| Maamba Collieries Ltd | ZCCM Investments Holdings Plc | 2014 | Zambia | Coal | 24 | 299 | 323 | 0.36 |
| Mazowe | Metallon Corporation Limited | 2014 | Zimbabwe | Gold | 795 | 1 066 | 1 861 | 2.05 |
| Namoya | Banro Corporation | 2014 | Dem. Rep. Congo | Gold | 34 | 39 | 73 | 0.08 |
| Otjikoto | B2Gold Corporation | 2014 | Namibia | Gold | 9 | 31 | 40 | 0.04 |
| Otjozondu | Rolek Resources Limited | 2014 | Namibia | Manganese | 33 | 43 | 76 | 0.08 |
| Bea Mountain | Avesoro Resources Inc. | 2015 | Liberia | Gold | 437 | 2 270 | 2 707 | 2.98 |
| Buckreef | Tanzanian Gold Corporation | 2015 | Tanzania | Gold | 200 | 983 | 1 183 | 1.30 |
| Kapulo | Mawson West Limited | 2015 | Dem. Rep. Congo | Copper | 38 | 228 | 266 | 0.29 |
| Trident - Sentinel | First Quantum Minerals Limited | 2015 | Zambia | Copper | 45 | 148 | 193 | 0.21 |
| Baobab | Avesoro Resources Inc. | 2016 | Senegal | Phosphate | 3 404 | 3 687 | 7 091 | 7.80 |
| Karma | Endeavour Mining Corporation | 2016 | Burkina Faso | Gold | 898 | 1 018 | 1 916 | 2.11 |
| Kokoya | Avesoro Resources Inc. | 2016 | Liberia | Gold | 353 | 762 | 1 115 | 1.23 |
| Nampala | Robex Resources Inc. | 2016 | Mali | Gold | 663 | 443 | 1 106 | 1.22 |
| Ancuabe | AMG Advanced Metallurgical Group N.V. | 2017 | Mozambique | Graphite | 40 | 296 | 336 | 0.37 |
| Balama | Syrah Resources Limited | 2017 | Mozambique | Graphite | 61 | 331 | 392 | 0.43 |
| Balogo | Avesoro Resources Inc. | 2017 | Burkina Faso | Gold | 392 | 459 | 851 | 0.94 |
| Fekola | B2Gold Corporation | 2017 | Mali | Gold | 242 | 242 | 484 | 0.53 |
| Franceville | Nouvelle Gabon Mining | 2017 | Gabon | Manganese | 43 | 290 | 333 | 0.37 |
| Gakara | Rainbow Rare Earths Limited | 2017 | Burundi | Lanthanides | 1 142 | 6 940 | 8 082 | 8.89 |
| Hounde | Endeavour Mining Corporation | 2017 | Burkina Faso | Gold | 388 | 377 | 765 | 0.84 |
| Yanfolila | Hummingbird Resources Plc | 2017 | Mali | Gold | 409 | 353 | 762 | 0.84 |
| Bel Air | Alufer Mining Limited | 2018 | Guinea | Bauxite | 486 | 471 | 957 | 1.05 |
| Boungou | SEMAFO Inc. | 2018 | Burkina Faso | Gold | 247 | 181 | 428 | 0.47 |
| Dian Dian | United Company RUSAL Plc | 2018 | Guinea | Bauxite | 596 | 636 | 1 232 | 1.35 |
| Imperial | Noble Group Limited | 2018 | Nigeria | Zinc | 340 | 606 | 946 | 1.04 |
| Mako | Resolute Mining Limited | 2018 | Senegal | Gold | 398 | 922 | 1 320 | 1.45 |
| Miniere Musoshi Kinsenda | Jinchuan Group International Resources Co. Ltd | 2018 | Dem. Rep. Congo | Copper | 59 | 364 | 423 | 0.47 |
| Rukwa Coalfield | Edenville Energy Plc | 2018 | Tanzania | Coal | 227 | 403 | 630 | 0.69 |
| Chirodzi | Jindal Steel & Power Limited | 2019 | Mozambique | Coal | 75 | 102 | 177 | 0.19 |
| Wahgnion | Teranga Gold Corporation | 2019 | Burkina Faso | Gold | 713 | 435 | 1 148 | 1.26 |
