# Supplementary material for: Assessing the effects of mining projects on child health in sub-Saharan Africa: a multi-country analysis
Source: Global Health. 2022 Jan 31;18:7. doi: 10.1186/s12992-022-00797-6 (PMC8802519; doi:10.1186/s12992-022-00797-6)
Supplement: Supplementary file 2 — Additional file 2 Table A2. Descriptive statistics of mine projects, including hosting country and primary commodity. [file 12992_2022_797_MOESM2_ESM.docx]

**Descriptive statistics for morbidity and the anthropometric dataset**

The final child morbidity and the anthropometric dataset contains a subset of data from 63 cross-sectional DHS datasets from 18 out of 34 SSA countries (53%% coverage). A total of 79 962 children from 40 983 households around 59 mining projects were included.

The descriptive statistics for child, mother and household-level characteristics are presented in Table A1. Most children (76%; N=60 753) were recorded after mine activation. Among children recorded after mine activation, 4.4% (N=2 682) lived close to active mines, which is similar to those recorded before mine activation. Table A1 also shows differences between comparison and impacted groups in pre and post-mine activation periods.

Data on childhood diarrhoea and cough were missing in 25 and 27 per cent of cases, respectively. For anthropometric measures, data were missing in more than 55% of recorded children. Missing data are due to the inclusion of non-standard DHS survey data in our primary dataset, such as the Malaria Indicators Survey (MIS) and Service Provision Assessment (SPA) surveys that do not record the above outcomes.

Overall, diarrhoea and cough prevalence is lower in children living closer to active mining projects than their peers living far away. In all groups, the mean anthropometric measures are above the benchmarks for child malnutrition.

**Table A1. Descriptive statistics for selected maternal and child morbidities and anthropometrics**

| **Variables** | **Total**  n = 79 962 | **Survey before mine activation**  **(n = 19 209)** | | **Survey after mine activation**  **(n = 60 753)** | |
| --- | --- | --- | --- | --- | --- |
|  |  | Impacted  [0-10 km] | Comparison  [10-50 km] | Impacted  [0-10 km] | Comparison  [10-50 km] |
|  |  | n = 856 | n = 18 353 | n = 2 682 | n = 58 071 |
| **Childhood morbidities** | | | | | |
| Child had diarrhoea recently ^¥,a^ | 10 132 (16.9%) | 136 (18.7%) | 2 958 (18.8%) | 226 (12.6%) | 6 812 (16.3%) |
| Child had cough recently ^¥,b^ | 13 321 (22.7%) | 191 (28.2%) | 4 281 (29.5%) | 354 (19.7%) | 8 495 (20.4%) |
| **Child anthropometrics** | | | | | |
| Height-for-Age z-scores: n (mean; sd) ^¥,c^ | 35 027 (-1.3; 1.7) | 243 (-1.1; 1.9) | 6 162 (-1.4; 1.8) | 1 118 (-1.4; 1.7) | 27 504 (-1.3; 1.7) |
| Weight-for-Age z-scores: n (mean; sd) ^¥,d^ | 35 609 (-1.0; 1.3) | 253 (-1.0; 1.7) | 6 359 (-1.1; 1.5) | 1 110 (-0.9; 1.3) | 27 887 (-1.0; 1.3) |
| Weigh-for-Height z-scores: n (mean; sd) ^¥,e^ | 34 594 (-0.3; 1.4) | 233 (-0.2; 1.8) | 6 143 (-0.4; 1.5) | 1 079 (-0.1; 1.3) | 27 139 (-0.3; 1.3) |
| **Child characteristics** | | | | | |
| Child is male | 40 589 (50.8%) | 410 (47.9%) | 9 289 (50.6%) | 1 354 (50.5%) | 29 536 (50.9%) |
| Child is single birth | 77 023 (96.3%) | 822 (96.0%) | 17 660 (96.2%) | 2 595 (96.8%) | 55 946 (96.3%) |
| Child age (months): n (mean; sd) ^¥,f^ | 73 877 (28.2; 17.3) | 756 (27.8; 17.3) | 16 318 (26.6; 17.1) | 2 529 (28.6; 17.6) | 54 274 (28.7; 17.2) |
| Birth order of a child: n (mean; sd) | 79 962 (3.5; 2.4) | 856 (3.8; 2.5) | 18 353 (4.0; 2.6) | 2 682 (3.4; 2.3) | 58 071 (3.4; 2.3) |
| **Maternal characteristics** | | | | | |
| Mother´s age (years): n (mean; sd) | 79 962 (29.1; 7.0) | 856 (28.7; 7.3) | 18 353 (29.0; 7.2) | 2 682 (29.1 (6.7) | 58 071 (29.1; 7.0) |
| Mother no education | 47 209 (59.0%) | 573 (66.9%) | 13 279 (72.4%) | 1 480 (55.2%) | 31 877 (54.9%) |
| Mother primary education | 17 915 (22.4%) | 154 (18.0%) | 2 994 (16.3%) | 626 (23.3%) | 14 141 (24.4%) |
| Mother secondary and higher education | 14 833 (18.6%) | 129 (15.1%) | 2 080 (11.3%) | 576 (21.5%) | 12 048 (20.8%) |
| Mother born less than 5 children | 51 028 (63.8%) | 544 (63.6%) | 10 839 (59.1%) | 1 790 (66.7%) | 37 855 (65.2%) |
| **Household (HH) characteristics** | | | | | |
| HH wealth: poorest quintile | 14 760 (18.7%) | 207 (24.2%) | 3 039 (17.4%) | 477 (17.8%) | 11 037 (19.0%) |
| HH wealth: second poorest quintile | 16 150 (20.4%) | 182 (21.3%) | 3 552 (20.3%) | 541 (20.2%) | 11 875 (20.5%) |
| HH wealth: third quintile | 15 698 (19.9%) | 129 (15.1%) | 3 492 (20.0%) | 578 (21.6%) | 11 499 (19.8%) |
| HH wealth: fourth quintile | 15 465 (19.6%) | 204 (23.8%) | 3 485 (20.0%) | 609 (22.7%) | 11 167 (19.2%) |
| HH wealth: fifth quintile (richest) | 16 999 (21.5%) | 134 (15.7%) | 3 895 (22.3%) | 477 (17.8%) | 12 493 (21.5%) |
| HH location is rural | 54 503 (68.2%) | 694 (81.1%) | 12 569 (68.5%) | 2 022 (75.4%) | 39 218 (67.5%) |

¥ Live children only; sd – standard deviation

^a^ missing data is 25.1%.

^b^ missing data is 26.7%.

^c^ missing data is 56.2%.

^d^ missing data is 55.5%.

^e^ missing data is 56.7%.

Figures are stratified by time to mine activation (i.e., ten years before the extraction period) and the DHS cluster´s distance to the mining sites. Data from 72 Demographic and Health Surveys from 18 SSA countries. The included DHS data was collected between 1992 and 2018 and restricted to clusters within 50 km from isolated mines (i.e., mines separated at a minimum distance of 20 km from each other). All measures represent unweighted sample proportions.
